# Comprehensive proteomic analysis of developing protein bodies in maize (*Zea mays*) endosperm provides novel insights into its biogenesis

**DOI:** 10.1093/jxb/erw396

**Published:** 2016-10-27

**Authors:** Guifeng Wang, Gang Wang, Jiajia Wang, Yulong Du, Dongsheng Yao, Bilian Shuai, Liang Han, Yuanping Tang, Rentao Song

**Affiliations:** ^1^Shanghai Key Laboratory of Bio-Energy Crops, School of Life Sciences, Shanghai University, Shanghai 200444, P.R. China, and; ^2^Coordinated Crop Biology Research Center, Beijing 100193, P.R. China

**Keywords:** Endosperm, prolamin, protein body, proteomic, storage protein, *Zea mays*.

## Abstract

Prolamins, the major cereal seed storage proteins, are sequestered and accumulated in the lumen of the endoplasmic reticulum (ER), and are directly assembled into protein bodies (PBs). The content and composition of prolamins are the key determinants for protein quality and texture-related traits of the grain. Concomitantly, the PB-inducing fusion system provides an efficient target to produce therapeutic and industrial products in plants. However, the proteome of the native PB and the detailed mechanisms underlying its formation still need to be determined. We developed a method to isolate highly purified and intact PBs from developing maize endosperm and conducted proteomic analysis of intact PBs of zein, a class of prolamine protein found in maize. We thus identified 1756 proteins, which fall into five major categories: metabolic pathways, response to stimulus, transport, development, and growth, as well as regulation. By comparing the proteomes of crude and enriched extractions of PBs, we found substantial evidence for the following conclusions: (i) ribosomes, ER membranes, and the cytoskeleton are tightly associated with zein PBs, which form the peripheral border; (ii) zein RNAs are probably transported and localized to the PB–ER subdomain; and (iii) ER chaperones are essential for zein folding, quality control, and assembly into PBs. We futher confirmed that OPAQUE1 (O1) cannot directly interact with FLOURY1 (FL1) in yeast, suggesting that the interaction between myosins XI and DUF593-containing proteins is isoform-specific. This study provides a proteomic roadmap for dissecting zein PB biogenesis and reveals an unexpected diversity and complexity of proteins in PBs.

## Highlight

Proteomic analysis of developing maize protein bodies (PBs) reveals an unexpected diversity and complexity of PB proteins, and provides a roadmap for the transport and translation of mRNAs of zein genes, and the assembly of PBs.

## Introduction

Cereals are the most important crops worldwide, with the annual production of maize, rice, and wheat grain totaling nearly 2500 million tonnes in 2013 (FAO, http://faostat3.fao.org/home/E). Unlike most dicots, the cereal endosperm is not ephemeral but is retained for the deposition of storage compounds during seed formation. The storage compounds in the endosperm are mainly composed of carbohydrates and proteins, which are essential for embryo development and germination, and are harvested for consumption by humans and livestock, and for industrial uses (Wang *et al.*, 2012*b*).

Compared to the overwhelming accumulation of starch, seed storage proteins on average account for only 10–12% dry weight of cereal grains (Shewry and Halford, 2002). According to their solubility, seed storage proteins can be classified into three major groups: the monomeric and water-soluble albumins, the homo-trimetric or hexametric and saline-soluble globulins, and the heterogeneous alcohol-soluble prolamins (Shewry and Halford, 2002; Kawakatsu and Takaiwa, 2010). Storage proteins are synthesized through the secretory pathway and deposited in different subcellular compartments. Water-soluble albumins and globulins are trafficked to protein storage vacuoles (PSV) progressively through Golgi-dependent vesicles (Herman and Larkins, 1999; Vitale and Ceriotti, 2004). In contrast, alcohol-soluble prolamins are sequestered and accumulated in the lumen of the endoplasmic reticulum (ER), and are directly assembled into protein bodies (PBs) (Larkins and Hurkman, 1978; Lending and Larkins, 1989; Vitale and Ceriotti, 2004).

In maize, prolamins, referred to as zeins, account for 70% of total endosperm proteins and are classified into α-, β-, γ-, and δ-zeins, each with distinct amino acid composition and structural properties (Shewry and Halford, 2002). The overall protein quality of maize kernels is quite poor due to the lack of the essential amino acids Lys and Trp in zeins. This nutritional deficiency is significantly improved in many opaque endosperm mutants, such as *o2* and *o7*, that display smaller and misshapen PBs and a reduced number overall (Schmidt *et al.*, 1990; Wang *et al.*, 2011). Concomitantly, however, these opaque mutants show susceptibility to insect and fungal damage, reduced yield, and many other adverse agricultural traits. The PB-inducing fusion system has been successfully applied to produce therapeutic and industrially important proteins in plants (Conley *et al.*, 2011). Thus, it is relevant to unravel the mechanisms underlying the assembly of zeins into PBs.

PB biogenesis in maize is precisely regulated at both the transcriptional and translational levels, including the spatial-temporal expression of individual zein genes, the direct interaction between different types of zeins, and the tight association with the actin-based actomyosin system (Woo *et al.*, 2001; Kim *et al.*, 2002; Wang *et al.*, 2012*a*). Zeins are highly packaged as discretely layered accretions in the ER lumen (Lending and Larkins, 1989; Holding *et al.*, 2007; Holding, 2014). PBs begin as small aggregations totally filled with γ-zeins, suggesting that γ-zeins are responsible for PB initiation. Indeed, 27-kDa γ-zein RNAi lines exhibit a drastically reduced number of PBs, but with normal size (Wu and Messing, 2010; Guo *et al.*, 2013). Further data indicates that 27-kDa γ-zein is retained at the ER through its N-terminal region that harbors eight Pro-rich repeats and seven Cys residues, possibly evolved from the storage-protein sorting to vacuoles pathway (Llop-Tous *et al.*, 2010; Mainieri *et al.*, 2014). In contrast, other γ-zeins (16- and 50-kDa) and 15-kDa β-zein RNAi lines have normal PB number but with reduced size, indicating that these γ- and β-zeins are necessary for PB filling but not initiation (Wu and Messing, 2010; Guo *et al.*, 2013). Simultaneous reductions in both 19- and 22-kDa α-zeins severely decreased PB size but did not induce other morphological abnormalities (Guo *et al.*, 2013). Interestingly, substantial suppression of only 22-kDa α-zeins or accumulation of either defective 19- or 22-kDa α-zeins resulted in small and misshapen PBs (Coleman *et al.*, 1997; Kim *et al.*, 2006; Wu and Messing, 2010; Wang *et al.*, 2014). Immunolocalization evidence indicates that 19-kDa α-zeins fill in the PB core while 22-kDa α-zeins preferentially deposit as an intermediary layer between the central 19-kDa α-zeins and the γ-zeins at the periphery, most likely controlled by *FL1* (Holding *et al.*, 2007). Together, the data suggest that each type of zein has a non-redundant function and that their proper stoichiometric ratio is essential for maintaining normal PB morphology (Guo *et al.*, 2013).

However, the protein composition of the native maize endosperm PB and the detailed mechanisms underlying its formation are still largely unknown. Here, the first comprehensive proteomic analysis was performed to isolate proteins involved in maize endosperm PB formation. This has identified 1756 candidate proteins that fall into several different categories, revealing an unexpected complexity of the native maize endosperm PB. Further analysis indicated that ribosomes, membranes, the cytoskeleton, and ER chaperones, as well as localization of zein mRNAs, play critical roles in the assembly of zeins into PBs in maize endosperm cells. In addition, we confirmed that there is no direct interaction between O1 (myosin XI) and FL1 (DUF593-containing protein).

## Materials and methods

### Plant material

Plants of maize inbred line B73 were cultivated in the field at the campus of Shanghai University. Developing kernels were collected at 20 d after pollination (DAP). Tobacco (*Nicotiana benthamiana*) plants were grown in a greenhouse under a 16/8 h day/night regime at a temperature of 20–25°C. Maize callus culture was established from scutellum and endosperm explants isolated from immature seeds of the hybrid Hi II line (Hi II pA × Hi II pB) and was maintained on Murashige and Skoog medium supplied with 2,4-D, as described previously (Šamaj *et al.*, 1999).

### Purification of PBs from developing endosperm

PBs were isolated using a novel strategy involving several rounds of discontinuous and continuous sucrose-density gradient centrifugation. All steps were performed at 4 °C. Fresh kernels (8 g) at 20 DAP were ground into powder in liquid nitrogen and then homogenized in 10 ml of 5 mM EDTA or 5 mM MgCl_2_ in suspension buffer (10 mM Tris-HCl pH 8.5, 10 mM KCl, 7.2% w/v sucrose, to which was added 1 mM DTT, 1 mM PMSF, and Roche Complete Protease Inhibitor cocktail before use) on ice for 15 min (Larkins and Hurkman, 1978). The homogenate was subsequently centrifuged for 10 min at 300 *g* to remove cellular debris. The supernatant (3.5 ml) was separated on a discontinuous sucrose-density gradient (1.0–1.5–2.0 mol l^–1^, 1:1:1, 3 ml each) with centrifugation at 35 100 rpm for 1 h (Habben *et al.*, 1993). The resulting interface of 1.5–2.0 mol l^–1^ was carefully recovered with a pipette and separated repeatedly with the same sucrose-density gradient and centrifugation parameters. The fraction of 1.5–2.0 mol l^–1^ was collected and pooled, and termed as the crude PBs (Sample A). The crude PBs were further purified using a multiple discontinuous sucrose-density gradient (40–45–50–55–60%, 1:1:1:1:1, w/w, 1.5 ml each) with centrifugation at 35 100 rpm for 1 h, and the interface of 50–55% was collected and separated again using the same multiple discontinuous sucrose-density gradient centrifugation. The collected 50–55% fraction was pooled, and termed as the enriched PBs (Sample B). This sample was then dispersed in a 10-ml continuous sucrose-density gradient (30–60%, w/w) to remove other possible contaminated organelles, with centrifugation at 35 100 rpm for 1 h. Fractions of 600 μl were collected systematically from top to bottom of the gradient. Then a mixture of fractions 13 to 15 was purified repeatedly under the same 30–60% (w/w) continuous sucrose-density gradient at 35 100 rpm overnight. Fractions of 600 μl were again collected and fraction 14 was pooled to obtain the highly purified intact PBs (Sample C). All pooled samples were diluted 2-fold with dilution buffer (10 mM Tris-HCl pH 8.5, 10 mM KCl) before the next centrifugation steps. The final collected PBs (Samples A, B, and C) were diluted 10-fold with the dilution buffer, and pelleted at 15 000 rpm for 10 min to remove sucrose. The pellet was resuspended in 10 ml of suspension buffer without sucrose and centrifuged at 13 000 rpm for 15 min. After discarding the supernatant, the PB pellet was dissolved in extraction buffer (0.0125 M sodium borate pH 10.0, 1% w/v SDS, 2% v/v 2-mercaptoethanol, and Roche Complete Protease Inhibitor cocktail), and then ethanol was added to a final concentration of 70% (Wallace *et al.*, 1990). After incubation and centrifugation, the resulting non-zein pellet from the PBs was frozen with liquid nitrogen and stored at –80 °C.

### Sample preparation and HPLC-MS

PB pellets were dissolved in 8 M urea supplied with 4% (w/v) CHAPS and 50 mM NH_4_HCO_3_. After acetone precipitation, the pellet was resuspended in the above solution and quantified using the Pierce^TM^ BCA Protein Assay Kit (Thermo Scientific). Protein was reduced by adding 0.1M dithiothreitol in 200 mM NH_4_HCO_3_ for 30 min at 56 °C and then alkylated by adding 0.5M iodoacetamide in 200 mM NH_4_HCO_3_ for 20 min at room temperature in the dark. The protein sample was finally digested using trypsin at a mass ratio of 1:50 enzyme/protein overnight at 37 °C. Digested peptides were resuspended in 0.1% (w/v) formic acid and insoluble components were removed by centrifugation at 12 000 rpm for 15 min. Samples (2 μg) were separated by liquid chromatography (LC) Easy-nLC 1000 (Thermo Scientific) with a C18 column (75 μm inner-diameter, 360 μm outer-diameter × 15 cm, 3 μm C18) and analyzed on an Orbitrap Elitemass spectrometer (Thermo Scientific), according to the manufacturer’s guidelines. The resulting MS spectra were opened with Analyst QS 2.0 software, and exported to MASCOT (Matrix Science, USA).

### Data analysis and bioinformatics

MASCOT was used to search against the maize genome database from MaizeGDB (http://ftp.maizegdb.org/MaizeGDB/FTP/), with an e-value ≤0.05. Coverage was calculated based on coverage of the complete protein sequence by matched peptide queries.

Information on protein function was compiled from the maize genome annotation and from the top five matches in the Arabidopsis genome using BlastP. Proteins were classified using gene ontology (GO) AmiGO2 with an e-value ≤0.05 (http://amigo.geneontology.org/amigo). Biological pathways analysis was performed utilizing the Kyoto Encyclopedia of Genes and Genomes (KEGG) Orthology-Based Annotation System v.2 (http://kobas.cbi.pku.edu.cn) (Xie *et al.*, 2011). Subcellular localization prediction was carried out using Target P (http://www.cbs.dtu.dk/services/TargetP/) and WoLFPSORT (http://cbrc3.cbrc.jp/cbrc/news/wolf_eng.html), as well as information for best-hit Arabidopsis homologs in published organelle proteomes in the Plant Proteome Database (PPDB) (http://ppdb.tc.cornell.edu/; Sun *et al.*, 2009). The abundance of the identified proteins was calculated using a normalized spectral abundance factors (NSAFs) method (Yuan *et al.*, 2014).

### Western blotting

Proteins were precipitated using the chloroform/methanol method. Immunoblotting was carried out as described previously (Wang *et al.*, 2012*a*), using antibodies against ER BiP (at-95, Santa Cruz Biotechnology, 1/1000), peroxisome PEX14P (Agrisera AS08 37, 1/10000), mitochondria IDH (Agrisera AS06203A, 1/5000), ribosome L13 (Agrisera AS132650, 1/2500), Golgi COPI (Agrisera AS08 327, 1/1000), and 15-kDa zein PBs (1/500) (Wang *et al.*, 2012*a*). Immunolocalization was performed as previously described (Kim *et al.*, 2006; Wang *et al.*, 2014), using an antibody against a randomly selected protein (GRMZM2G346263_P03, 1/200) for localization validation, which was produced by Shanghai ImmunoGen Biological Technology in rabbits according to standard protocols.

### RNA extraction and constructs

Immature kernel RNA was isolated using a protocol described previously (Wang *et al.*, 2012*c*). After removing the residual DNA by an RNase-free DNaseI (Takara) treatment, 2 μg total RNA was reverse-transcribed to cDNA using RevertAid H Minus Reverse Transcriptase (Thermo). cDNAs were amplified by PCR using KOD plus polymerase (Toyobo) using primers listed in Supplementary Table S1 at *JXB* online, and then were cloned into pENTR/D-TOPO using the Gateway TOPO cloning kit (Invitrogen) and sequenced. The verified entry clones were introduced into the corresponding destination vector pB7CWG2.0 for subcellular localization by the LR reaction of the Gateway system (Invitrogen). The binary expression vectors were transformed into *Agrobacterium tumefaciens* strain GV3101.

### Transient expression and confocal laser scanning microscopy

A transient expression assay was performed using a method described previously (Reyes *et al.*, 2010; Gao *et al.*, 2013). Briefly, 4-week-old tobacco plants and fresh maize calli were used for the transient assays. *Agrobacterium* cells transformed with plasmids harboring a cyan fluorescent protein (CFP) fusion gene of interest were infiltrated into plant tissues. For detection of PB fractions, the dilute samples were incubated with ER tracker red dye (Molecular Probes, ThermoFisher) and rhodamine B hexyl ester (Molecular Probes, ThermoFisher) according to the manufacturer’s instructions. Images were obtained using a Zeiss confocal microscope LSM 710 (Carl Zeiss). We used 458-, 587-, and 528-nm laser excitation and 460–510,600–630, and 527–570 nm long-pass emission filters to detect CFP, ER tracker red dye, and rhodamine B hexyl ester, respectively. All images were analyzed using the Image J software (https://imagej.nih.gov/ij/).

### Yeast two-hybrid assay

cDNA fragments were generated by PCR and ligated into the pMD18-T vector (Takara). After sequence confirmation, they were fused downstream to either the GAL4BD domain in pGBKT7 or the GAL4AD domain in pGADT7 at the appropriate enzyme sites listed in Supplementary Table S1. Yeast transformation and screening procedures were performed according to the manufacturer’s instructions (Clontech). Yeast strain Y187 was co-transformed with pGBKT7-baits and pGADT7-preys. The human P53 and maize 50-kDa zein were used as positive controls. The putative positive clones were further spotted with a series of dilutions on SD/-Ade/-His/-Leu/-Trp medium.

## Results and discussion

### Distribution profiles of major organelles in developing maize seeds under a 30–60% (w/w) continuous sucrose-density gradient sub-fractionation

The spatial-temporal expression of zein genes is primarily controlled at the transcriptional level, and the proteins generally accumulate during the middle and late stages of kernel development (Woo *et al.*, 2001; Wang *et al.*, 2012*a*). Thus, PBs were isolated from developing kernels at 20 DAP when they are more abundant and nearly mature. To determine the localization of different organelles, we employed a 30–60% (w/w) continuous sucrose-density gradient to sub-fractionate them from the 20 DAP maize developing kernels. After centrifugation, 16 fractions (600 μl each) were collected from top to bottom of the linear gradient. Silver-stained analytical SDS gels confirmed that zeins are the major proteins deposited in maize kernels and distribute predominantly in the fractions with much higher sucrose density (Supplementary Fig. S1A). Immunoblotting was performed to detect the presence of major organelles, using antibodies raised against markers including BiP (ER), PEX14P (peroxisome), IDH (mitochondria), L13 (ribosome), COPI (Golgi), and 15-kDa zein (PB).

**Fig. 1.**
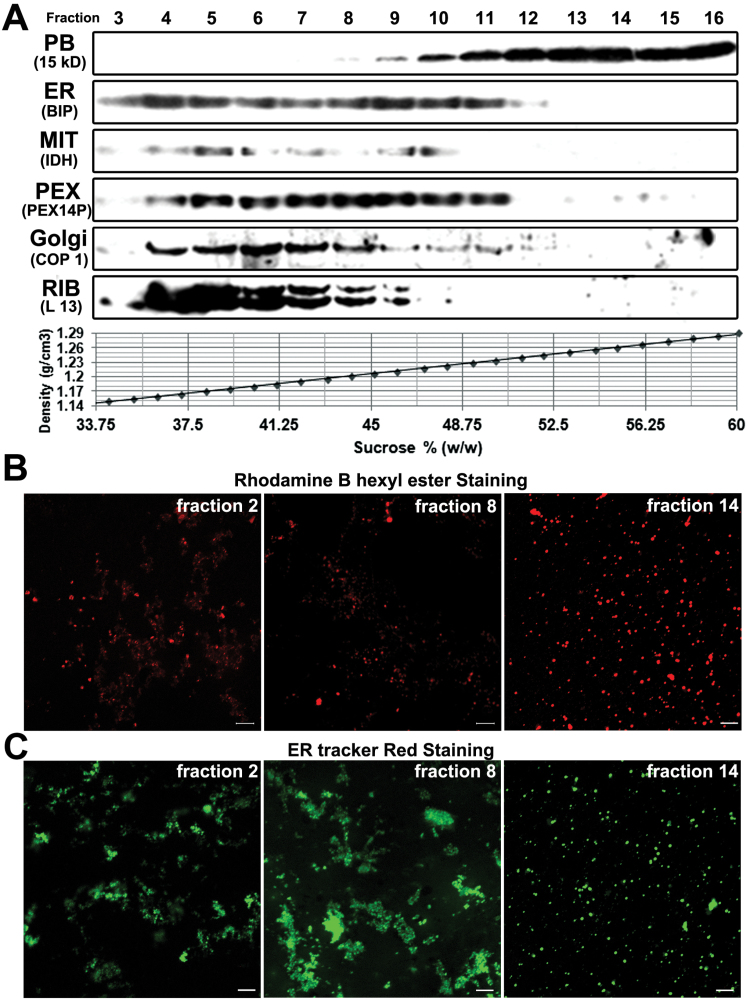
Distribution patterns of major organelles in maize seed 20 DAP under a 30–60% (w/w) continuous sucrose-density gradient sub-fractionation. (A) Western blotting for determining the presence of major organelles. Proteins from each fraction were precipitated using the chloroform/methanol method and subjected to immunoblotting using antibodies raised against markers including BiP (ER), PEX14P (peroxisome), IDH (mitochondria), L13 (ribosome), COPI (Golgi), and 15-kDa zein (PB). (B, C) The quality assessment of PBs from different fractions using immunostaining with the PB-specific dye rhodamine B hexyl ester (B) and ER tracker dye (C). The fraction number corresponds to that isolated from the 30–60% (w/w) continuous sucrose-density gradient centrifugation (fractions taken top to bottom). Scale bars =10 µm.

As shown in Fig. 1A, ER, peroxisomes, mitochondria, ribosomes, and Golgi bodies were mainly distributed into the fractions ranging from 3 to 11 (sucrose density 1.14–1.24 g cm^–3^), while PBs were abundantly present in fractions from 12 to 16 (sucrose density 1.25–1.29 g cm^–3^). We calculated and summarized the distribution range and density of major organelles in maize seeds under the 30–60% (w/w) continuous sucrose-density gradient (see Supplementary Table S2). Two conspicuous peaks appeared in the ER distribution area, corresponding to the smooth (1.15 g cm^–3^) and rough (1.22 g cm^–3^) ER. This result is consistent with previous reports in Arabidopsis and tobacco BY-2 cells (Ueda *et al.*, 2010; Yokota *et al.*, 2011). Compared to the PBs induced in tobacco leaves by transient transformation with Zera fused to DsRed (Joseph *et al.*, 2012), the maize endosperm PBs are highly compacted, with smaller sizes (1–2 to 2–2.5 µm) but much higher densities (1.25–1.28 to 1.21–1.23 g cm^–3^). Immunostaining with rhodamine B hexyl ester confirmed that PBs were abundant in the higher-density fractions (Fig. 1B). ER tracker staining further showed that the PBs in these fractions were highly purified, without detectable ER-surrounding structures (Fig. 1B, fraction 14). Together, the data indicated that the intact maize endosperm PBs are structurally and organizationally different from the PB-inducing fusions derived from other cellular systems, and could be easily discriminated from other organelles by using sucrose-density gradient centrifugation.

### A novel protocol for isolating highly purified, intact PBs in maize endosperm

**Fig. 2.**
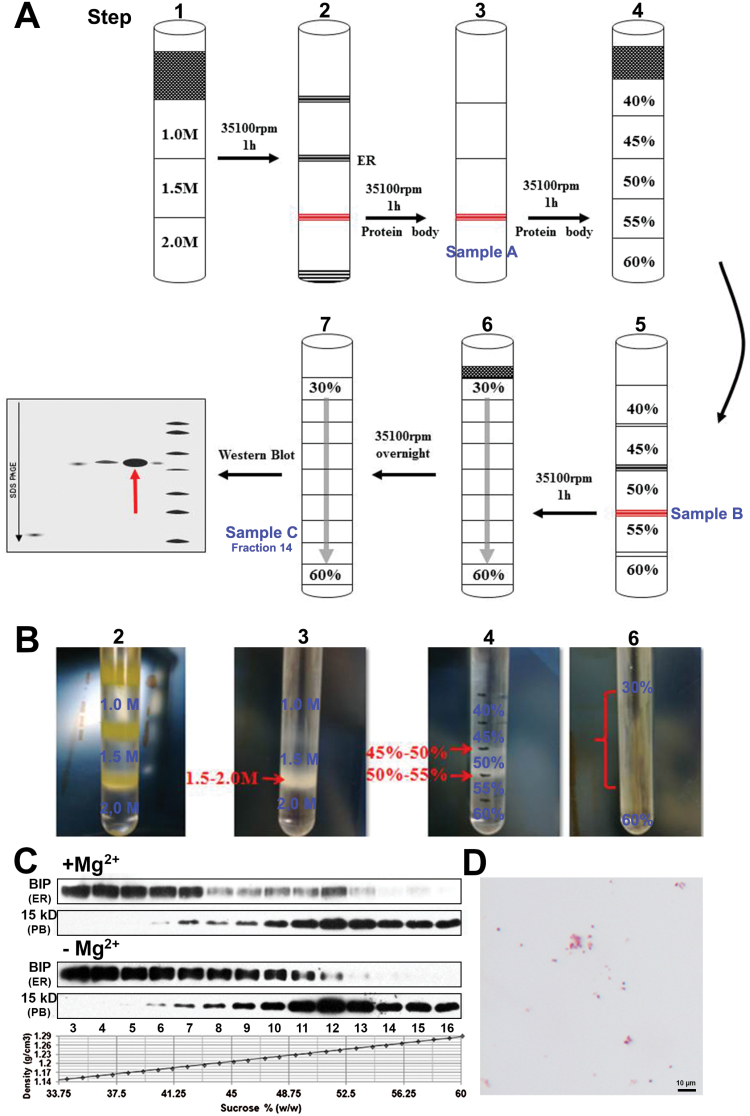
A novel approach for the isolation of highly purified intact PBs from maize endosperm. (A) Work flow of the stepwise method with several rounds of discontinuous and continuous sucrose-density gradient centrifugation. (B) Representative images of the different sucrose-density gradient centrifugation samples. The numbers correspond to the same steps labeled in (A). (C) Western blotting for determining the presence of PBs and ER using buffers with or without Mg^2+^. Proteins from each fraction were precipitated using the chloroform/methanol method and subjected to immunoblotting using antibodies raised against the markers 15-kDa zein (PB) and BiP (ER). (D) Light microscopic observation of the final purified PBs (Sample C) with fuchsin staining. (This figure is available in colour at *JXB* online.)

Previously, crude PBs have been successfully isolated from maize endosperm using the discontinuous sucrose-density gradient centrifugation (Habben *et al.*, 1993; Holding *et al.*, 2007). Because even minor contamination with other organelles would significantly affect the following proteomic analysis, a novel protocol that combined several rounds of discontinuous and continuous sucrose-density gradient centrifugation was therefore developed to isolate highly purified, intact PBs from maize endosperm (Fig. 2A, B).

Briefly, two rounds of conventional discontinuous sucrose-density gradient (1.0–1.5–2.0 mol l^–1^) centrifugation (Habben *et al.*, 1993) were first applied to extract the crude PBs from the 20-DAP maize kernels. The resulting PB fraction (the interface of 1.5–2.0 mol l^–1^, Fig. 2B) was purified using a multiple discontinuous sucrose-density gradient (40–45–50–55–60%, w/w), and the PB-rich fraction (interface of 50–55%, corresponding to 1.23–1.26 g cm^–3^) was further separated using the same multiple discontinuous sucrose-density gradient centrifugation. The collected 50–55% fraction was then dispersed in a 30–60% (w/w) continuous sucrose-density gradient to remove other possible contaminating organelles. Finally, a mixture of fractions 13 to 15 was purified repeatedly to get highly purified PBs (fraction 14), under the same 30–60% (w/w) continuous sucrose-density gradient. Silver-stained analytical SDS gel analysis indicated that only the zeins were detectable in the isolated PBs (Supplementary Fig. S1B). Since PBs are ER-derived organelles, they would be easily contaminated by cisternal ER and ER-bound ribosomes. EDTA was used to depolymerize ribosomes and cause ER migration. We found that the presence of EDTA could effectively decrease the ER density but not that of the PBs (Fig.2C; Supplementary Fig. S2). Light microscopy showed that the purified PBs were spherical with a diameter of 1–2 μm and almost detached from each other, without any observed contaminants (Fig. 2D). Thus, we have determined a method to isolate highly purified, intact PBs from developing maize endosperm, suitable for proteomic analysis.

### A total of 1756 proteins are identified in the highly purified intact PBs from the maize developing endosperm at 20 DAP

To evaluate reproducibility and differences, three different PB fractions were collected for subsequent study (Fig. 2A), namely the crude PBs (the interface of 1.5–2.0 mol l^–1^, Sample A), the enriched PBs (interface of 50–55%, Sample B), and the highly purified intact PBs (Sample C). Prior to trypsinization and HPLC-MS assay, zeins were removed using ethanol extraction in order to enrich the low-abundance proteins (Wallace *et al.*, 1990).

**Fig. 3.**
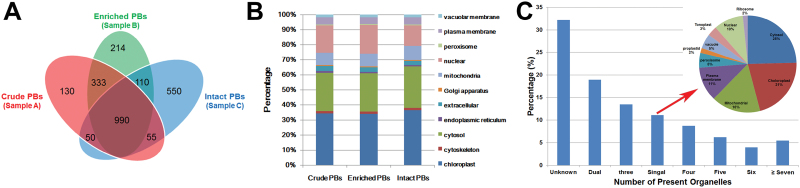
Characterization of proteins identified in maize endosperm PBs at 20 DAP. (A) Venn diagram comparing the presence of proteins identified from the three different PB samples: crude, enriched, and intact PBs. (B) Subcellular localization of the identified proteins in the three PB proteomes predicted by the WoLFPSORT program. (C) Prediction of subcellular localization of the 550 proteins unique to the intact PBs based on information for their best-hit Arabidopsis homologs on the subcellular proteomes in the PPDB (http://ppdb.tc.cornell.edu/). The ‘number of present organelles’ represents the number of the subcellular proteomes commonly identified for a given protein. For details, see the Supplementary Table S4. The inset indicates the localization of the proteins identified only in a single organelle proteome.

A total of 2432 unique proteins were obtained from 1558, 1647, and 1756 proteins identified in Samples A, B, and C, respectively (Supplementary Table S3; raw data in Supplementary Dataset S1). The majority (990) proteins were common to all three samples; 548 were found in any two of the three PB samples, and 895 were found in only one of the three samples (Fig. 3A). WoLFPSORT prediction revealed that the 2432 proteins could be mainly targeted to chloroplasts, the cytosol, the nucleus, and mitochondria (Fig. 3B), which is similar to a recent proteomic analysis of ER-derived PBs in tobacco leaves (Joseph *et al.*, 2012). Notably, the percentage identified with nuclear localization dramatically reduced while that in chloroplasts and the cytosol slightly increased in the highly purified intact PBs, relative to the other two PB samples. Of the 130 proteins unique to the crude PBs, the majority appeared to be contaminated by the ER secretory pathway, mitochondria and chloroplast (Supplementary Table S3). We further referenced the 550 proteins unique to the highly purified intact PBs against the subcellular proteomes in the PPDB (Sun *et al.*, 2009). One third of them produced no hits in the databases and more than half (56.7%) were present in at least two different organelles (Fig. 3C and Supplementary Table S4). These proteins are most likely localized in the maize endosperm PBs. Only 10% of them were identified in a single organelle proteome, most likely the contaminants mainly from the cytosol, chloroplasts, and mitochondria (inset in Fig. 3C). Besides zeins, the only two PB-associated proteins so far identified, O1 (GRMZM2G449909_P01, No. 1422) (Wang *et al.*, 2012*a*) and FL1 (GRMZM2G094532_P01, No. 262) (Holding *et al.*, 2007), were both present in the proteomes.

**Fig. 4.**
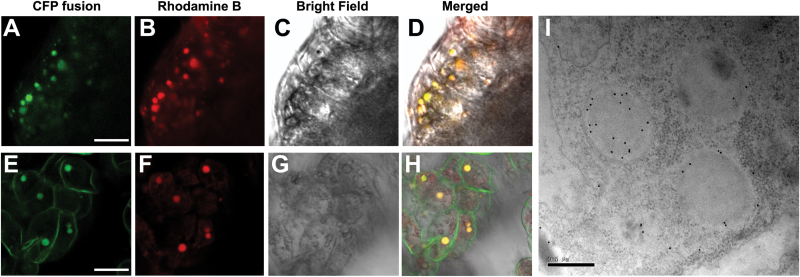
Validation of subcellular localization of three candidate proteins. Localization of two CFP-fusion proteins GRMZM2G091503_P04 (A–D) and GRMZM2G122267_P02 (E–H) in maize callus storage cell PBs stained with the rhodamine B hexyl ester. Confocal microscopic images were taken from maize callus storage cells using the *Agrobacterium*-mediated transformation method as described by Šamaj *et al.* (1999) and Reyes *et al.* (2010). Scale bars =10 µm. (I) Immunolocalization of GRMZM2G346263_P03 in the developing endosperm at 21 DAP. Scale bar =0.5 μm.

To further validate the targeting of the proteins present in the PB proteome, we investigated the subcellular localization of two randomly picked proteins (GRMZM2G091503_P04 and GRMZM2G122267_P02) using CFP fusion analysis driven by the 35S promoter. An *Agrobacterium*-mediated transient expression in maize callus storage cells was performed as described previously (Šamaj *et al.*, 1999; Reyes *et al.*, 2010). As shown in Fig. 4A–H, both proteins were confirmed to be targeted to maize PBs, largely with one-on-one co-localization to the staining of the PB-specific dye rhodamine B hexyl ester. Immunolocalization detection on ultrathin sections of developing maize kernels labeled with an antibody raised against an unknown protein (GRMZM2G346263_P03, No. 1535) identified in the PB proteome indicated that this protein was preferentially localized in PBs, with a small fraction also present in the ER (Fig. 4I). The abundance of the identified proteins among the three PB proteomes was calculated using a normalized spectral abundance factor (NSAF) method (Yuan *et al.*, 2014). The NSAF values of most ribosome- and ER-related proteins were severely decreased in the intact PBs relative to the crude PBs and the enriched PBs (Supplementary Tables S5 and S6). It should be mentioned that some potentially important proteins that were less tightly adhered to PBs could be lost using this step-wise purification procedure, as notably illustrated by the complete loss of PB-assembly factor O1 in the intact PBs. Collectively, these data indicated that the proteins identified in the intact PB proteome are highly reliable.

### The basic features of the intact developing maize endosperm PB proteome

**Fig. 5.**
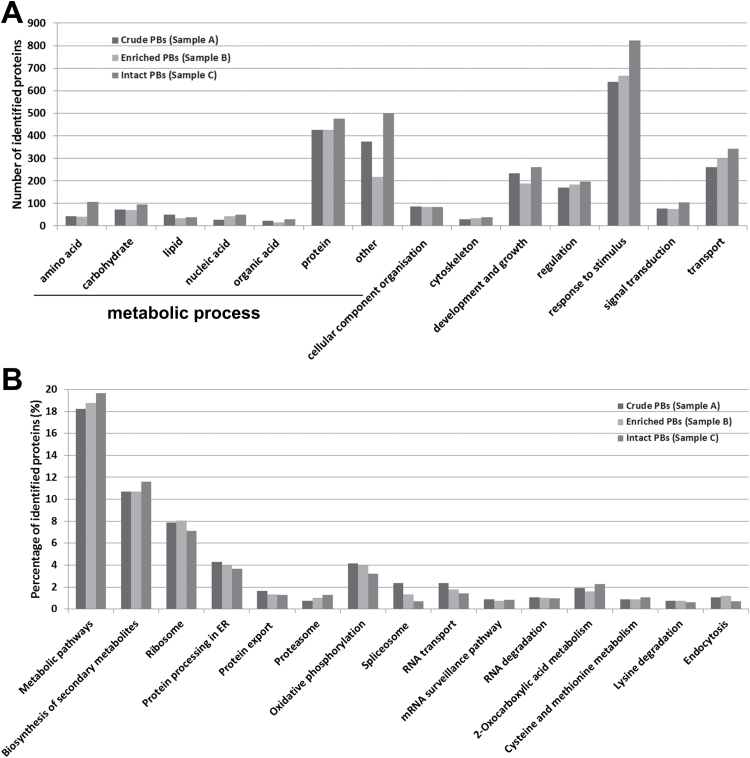
Functional classification of 2432 proteins identified in maize endosperm PBs. (A) Number of identified proteins grouped by the GO biological process analysis. (B) Percentage of proteins involved in different categories according to the KEGG database (http://www.genome.jp/kegg/).

With the aim of highlighting the biological processes underlying maize endosperm PB biogenesis, the identified proteins in the PB proteomes were queried using the AmiGO browser, and were largely found to be classified into 14 categories (*P*-value <0.05, FDR<0.05; Fig.5A and Supplementary Dataset S2). Notably, around two fifths of the proteins were clustered into metabolic process (GO: 0008152), of which 40% belonged to protein metabolism, including translation, protein processing, folding, and modification. The second largest group was related to response to stimulus (GO: 0050896), which contributed one quarter of the PB proteome. More than 10% of the identified proteins were involved in transport (GO: 0006810), suggesting an extensive intracellular trafficking during PB biogenesis. The remaining proteins were related to other biological categories, including development and growth, regulation, signal transduction, and cellular component organization. Similarly, the biological processes enriched in a recently reported maize non-zein proteome included protein transport, protein folding, proteolysis, and biosynthetic processes (Morton *et al.*, 2016). Noticeably, this non-zein proteome was derived from the whole kernels whereas our PB proteome is restricted to proteins tightly adhered to or resided in PBs.

The PB proteomes were further mapped to the metabolic pathways using the maize KEGG database (http://www.genome.jp/kegg-bin/show_organism?org=zma, *P*-value <0.05). As shown in Fig. 5B and Supplementary Dataset S3, metabolic pathways (ID: zma01100) was the largest category (18%), followed by biosynthesis of secondary metabolites (ID: zma01110, 10%), ribosome (ID: zma03010, 7%), protein processing in endoplasmic reticulum (ID: zma04141, 4%), and oxidative phosphorylation (ID: zma00190, 4%). In agreement with the NSAF values, the percentages of ribosome and ER categories were also reduced in the intact PB sample, compared to the other two samples.

### Ribosomes, ER membranes and the cytoskeleton are closely associated with zein PBs, forming the peripheral border

Previously, cytological and biochemical evidence has shown that PBs are enclosed by filamentous actin, polyribosomes, and the ER membrane (Larkins and Hurkman, 1978; Lending and Larkins, 1989; Stankovic *et al.*, 1993). To eliminate contamination by ER-bound ribosomes, we combined several rounds of purification and ribosome depolymerization by adding EDTA in order to get purified and intact maize endosperm PBs (Fig. 2C). Surprisingly, a total of 118 ribosome-associated proteins were still identified in the intact PB proteome, although their percentage and abundance were reduced relative to that in both the crude PBs and the enriched PBs (Fig.5B and Supplementary Table S5). These proteins represented most ribosome small (Sa-B, S3a, S4, S5, S7e, S8, S12, S13a, S17, S24e, S25, S26e, and S27a,) and large (P2, L1p/L10e, L4, L5-1, L6, L7Ae, L10, L11, L13, L14, L18e, L19e, L22p/L17e, L28e, and L38e) subunits, with at least two distinct members (Chang *et al.*, 2005). These data suggested that ribosomes are tightly associated with PBs and these PB-bound ribosome complexes are more robust than other protein complexes. Among the proteins involved in the secretary pathway, we identified many putative membrane proteins resident in the ER (Supplementary Table S6). FL1 is the first characterized ER membrane protein surrounding the PB, and affects PB biogenesis most likely by facilitating 22-kDa α-zein deposition (Holding *et al.*, 2007). We also identified an ortholog (GRMZM2G007871_P01, No. 1168) of the Arabidopsis J domain ER membrane protein AtERdj2A, which is essential for growth in Arabidopsis as it affects pollen germination (Yamamoto *et al.*, 2008). In addition, its yeast ortholog Sec63p is required for cell growth by mediating protein translocation across the ER membrane (Rothblatt *et al.*, 1989). Additionally, several integral membrane proteins were found, including four emp24/gp25L/p24 family/GOLD family members and three endomembrane protein 70 isoforms, both of which are implicated in sorting and packaging cargos for ER export via binding between the coated protein and their cytoplasmic domains (Anantharaman and Aravind, 2002; Gao *et al.*, 2012). Interestingly, 15 actin-related and 10 tubulin-associated cytoskeletal proteins were also identified in the intact PB proteome (Supplementary Table S3). These data support the hypothesis that the cortical actin filament and microtubule networks are involved in anchoring and maintaining the PB–polyribosome–ER membrane complex to the plasma membrane (Muench *et al.*, 2000). Taken together, our data indicated that ribosomes and ER membranes (probably derived from rough ER), as well as the cytoskeleton, although in the peripheral region, are most likely the integral compartments of zein PBs.

### The potential role of zein RNA transport and localization in maize endosperm PB biogenesis

In rice endosperm, storage protein prolamins and glutelins are synthesized in two specific ER subdomains, with prolamine mRNAs being localized to the PB and encased by the rough ER (PB-ER) whereas glutelin mRNAs are localized to the cisternal ER (Crofts *et al.*, 2004), and finally generating two different compartments PB-I and PB-II, respectively. A conserved mechanism has been proposed for localization of zein mRNAs, although only type I PBs are generated in maize endosperm (Washida *et al.*, 2004, 2009). Notably, a high proportion of proteins related to RNA metabolism were identified, including RNA splicing, transport, surveillance, and degradation (Fig. 5B). In particular, we found 40 RNA-binding proteins, 23 translation initiation factors, 16 putative translation elongation factors, and three Tudor family proteins (Supplementary Table S3), all of which are implicated in the RNA localization pathway (Martin and Ephrussi, 2009). We further compared these proteins with those RNA-binding proteins captured using prolamine zipcode bait RNA in rice endosperm cells (Crofts *et al.*, 2010). Seven out of the 16 ortholog pairs were found in the maize intact PB proteome, including two malate dehydrogenases (MDH, RBP-C and M), a cold-shock protein (RBP-H), a putative RNA-binding protein (RBP-L), 60S ribosomal L3 (RBP-O), a glycine-rich RNA-binding protein (RBP-R), and Tudor-SN (Supplementary Table S7). Interestingly, none of the heterogeneous nuclear ribonucleoprotein (HnRNP) orthologs were detected in the intact PB proteome, although some of them are likely to be involved in rice prolamine RNA localization. This is perhaps not surprising because HnRNPs predominantly shuttle between the nucleus and the cytosol to participate in mRNA biogenesis and mRNA export from the nucleus (Yeap *et al.*, 2014). Rice RBP-A, RBP-I, RBP-J, RBP-K, and RBP-Q were consistently distributed into both the nucleus and the cytoplasm (Yang *et al.*, 2014). In particular, RBP-A co-localized with the nucleus, the cisternal ER membrane, and microtubules but not with the PB-ER (Crofts *et al.*, 2010). This suggests that these HnRNPs might primarily function in export of prolamine RNAs from the nucleus and transport in the cytosol before their localization in the PB-ER. Moreover, they could form several protein complexes (A–J–K, I–J–K) on the prolamine zipcode from the nucleus to the cytoplasm (Yang *et al.*, 2014). It should be pointed out that two members (RBP-A and K) of the complexes have no corresponding orthologs in the maize genome. Thus, it seems that other HnRNPs are responsible for zein mRNAs transport and localization. Another cytoskeletal-associated RBP, Tudor-SN, could bind both prolamine and glutelin RNA-RNP particles and separately transport them into the PB-ER and cisternal ER in rice endosperm cells (Wang *et al.*, 2008). Downregulation of Tudor-SN resulted in decreased expression of prolamine genes at both transcriptional and translational levels, and thus a reduced number of prolamine PBs (Wang *et al.*, 2008). In *Caenorhabditis elegans*, *Drosophila*, and mammals, Tudor-SN was found to be a component of the RNA-induced silencing complex (RISC) and contributed to the target RNA degradation (Caudy *et al.*, 2003). It would be interesting to determine whether Tudor-SN performs a conserved function in maize zein mRNAs transport and translational quiescence during the transport process. Furthermore, we also found many RNA degradation-related proteins, which might be responsible for the decay of zein mRNAs after translation in the PB-ER.

### ER chaperones are essential for zein folding, quality control, and PB assembly

In the proteome of Zera-DsRed induced PBs, over one-third of the identified proteins were predicted to enter the ER secretory pathway (Joseph *et al.*, 2012). Similarly, we found around 4% of the proteins belonged to protein processing in the endoplasmic reticulum (ID: zma04141; Fig. 5B). Two isoforms of binding immunoglobulin proteins (BiPs) were among the most abundant proteins detected in our PB proteomes, but the abundance was significantly reduced in the intact PBs (Supplementary Table S6). BiPs, a hallmark of ER folding proteins, have been proposed to mediate rice prolamine PB biogenesis (Li *et al.*, 1993). Modulating the expression of BiP1 (increase or decrease) could alter the intracellular structure of endosperm cells and also reduce storage proteins and starch accumulation by alleviating ER stress (Wakasa *et al.*, 2011). In maize, it has also been shown that a BiP was localized in the peripheral regions of PBs and associated with PB morphogenesis (Zhang and Boston, 1992; Hunter *et al.*, 2002). We found ten DnaJ-domain and 13 other ER-resident HSP70 (heat shock protein) family members; the former could differentially interact with distinct BiPs and localize in both the ER and the outer regions of prolamine PBs in the rice endosperm (Ohta *et al.*, 2013). Disulfide bonds are also required for accumulation of storage proteins in cereal endosperm (Shimoni *et al.*, 1995; Onda *et al.*, 2011). Protein disulfide isomerase (PDI), the donor of disulfides, is involved in protein sorting in the ER through the thioldisulfide exchange reaction. Over ten different PDI isoforms were found in our PB proteomic analysis. Notably, we detected the putative orthologs of rice PDIL1;1 and PDIL2;3, which play distinct roles in sulfhydryl oxidations of structurally diverse storage proteins during rice PB formation (Onda *et al.*, 2011). Specifically, PDIL2;3 primarily localizes on the prolamine PB surface and is responsible for the deposition of Cys-rich 10-kDa prolamin in the core of prolamine PBs, whereas the ER lumen-localized PDIL1;1 is beneficial to the oxidative folding of vacuole-targeted proglutelins and α-globulin. One calreticulin, three calnexins, and other HSPs were present in our PB proteome. Taken together, it is most likely that the nascent zeins first correctly fold by binding to BiPs, calnexins, and other chaperones, and then aggregate to form polymers through the formation of intermolecular disulfide bonds between cysteine residues mediated by PDIs. Additionally, we found many putative membrane-spanning proteins (Supplementary Table S8), such as FL1 and an unknown PB-localized protein, which are potentially involved in shaping the ER membrane from the lumenal side after interaction with zeins.

An interesting but confusing issue is the protein quality control mechanism underlying zein retention and assembly into PBs in the ER lumen. Due to the lack of an ER retention signal in zein polypeptides, how they succeed in aggregating in the ER instead of being exported from the ER is uncertain. A convincing explanation is the hydrophobic nature of zeins that could effectively retard their inclusion in COPII vesicles (Vitale and Ceriotti, 2004). In the case of the 27-kDa γ-zein, its N-terminal region contains eight VHLPPP repeats and seven Cys residues that could form inter-chain disulfide bonds, which could be sufficient for ER retention (Llop-Tous *et al.*, 2010). Recently it has been shown that progressive substitution of the seven Cys into Ser residues in the 27-kDa γ-zein could gradually increase its solubility and traffic to the vacuole via a wortmannin-sensitive pathway, leading to the proposal that the deposition of zeins in the ER evolves from storage protein sorting to vacuoles (Mainieri *et al.*, 2014). Knock-down of the 27-kDa γ-zein resulted in severe reduction in PB number, but those remaining had normal size (Wu and Messing, 2010; Guo *et al.*, 2013; Yuan *et al.*, 2014). Altogether, these data suggest that the 27-kDa γ-zein is responsible for PB initiation through its N-terminus resident in the ER. Furthermore, a series of combinations of RNAi constructs of different zein classes showed that 16- and 50-kDa γ-zeins are involved in PB expansion and α-zeins are related to PB filling (Guo *et al.*, 2013). Noticeably, in the 27-kDa γ-zein RNAi, α-zeins deposited not only in the peripheral region of the PB but also in the ER membrane that was disconnected with the PB. This result indicates that α-zeins also could be retained in the ER independently of the interaction with γ-zeins (Kim *et al.*, 2002), probably with the aid of BiPs (Zhang and Boston, 1992). Even in some mutants with elevated ER stress, the defective zeins could still accumulate in the misshapen PBs and the ER membrane, rather than entering the protein quality control system and being degraded by the ER-associated degradation pathway (Kim *et al.*, 2006; Wang *et al.*, 2014). Therefore, ER chaperones play an essential role in zein folding, aggregation, and assembly into PBs, avoiding entry into the ER export pathway and degradation via the protein quality control system.

### The potential role of myosins XI and DUF593-containing proteins in maize endosperm PB biogenesis

Recently it has been shown that the direct interaction between myosin XI-K and the DUF593 domain-containing protein MyoB1 is responsible for transporting a distinct class of plant-specific vesicles in Arabidopsis (Peremyslov *et al.*, 2013). Interestingly, three myosins, ZmMyo11E3 (XI-K), ZmMyo11G1 (O1/XI-I), and ZmMyo11A1 (XI-B), were found in our crude PBs (Samples A and B) but were absent in the intact PB proteome; meanwhile two DUF593 proteins including FL1 were also detected (Supplementary Table S3). This suggests a potential interaction between O1 and FL1 that are corporately involved in maize PB formation, although by using the yeast two-hybrid (Y2H) assay we previously found that the O1-tail domain cannot interact with the complete FL1 (Wang *et al.*, 2012*a*).

**Fig. 6.**
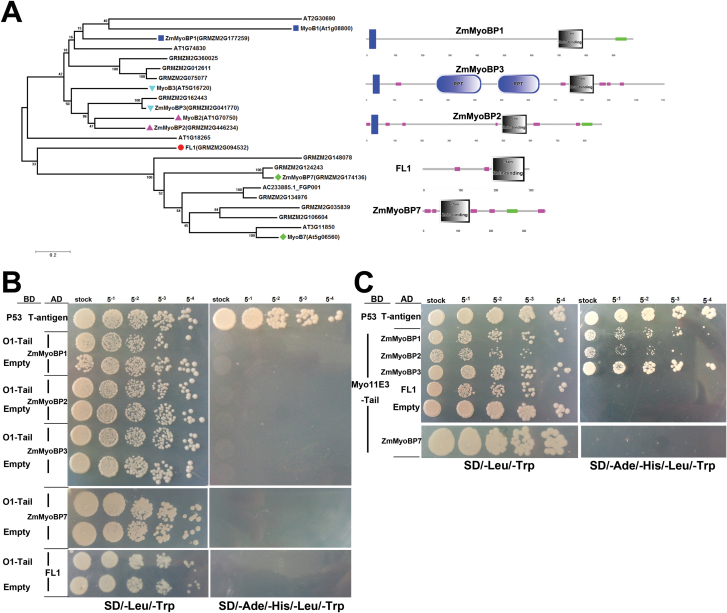
Interactions between mysion XI members and DUF593- (zein binding) containing proteins identified in the maize PB proteome. (A) Phylogenetic tree and domain architectures of DUF593-containing proteins. The evolutionary history was inferred using the Neighbor-Joining method provided in MEGA6 (http://mega6.software.informer.com/) on the basis of the multiple alignment of the full-length proteins from Arabidopsis (At) and maize (Zm). The same shapes indicate putative best-hit homologs. The panel on the right shows the domain arrangements of the maize DUF593 proteins. The large upright boxes at the N-terminal represents the transmembrane domain; the long horizontal bars at the C-terminal indicate the coiled-coil domain; the smaller horizontal boxes appearing throughout the length of the proteins represent low-complexity regions; RPTindicates repeat domains; Zein-binding represents zein-binding domains (DUF593). (B) Yeast two-hybrid assay interactions between the O1-tail domain and DUF593 domains of different members. (C) Interaction between the Myo11E3-tail and DUF593 domains. Interaction between T-antigen and P53 was used as positive control. AD, activating domain; BD, binding domain. (This figure is available in colour at *JXB* online.)

To test the putative conserved interactions between different myosin XI and DUF593 members in maize, a neighbor-joining phylogenetic tree was constructed using the full-length DUF593 domain-containing proteins from Arabidopsis and maize. As shown in Fig. 6A, FL1 is divergent from the MyoB7 subgroup, while another identified DUF593 protein (GRMZM2G174136) is highly homologous to Arabidopsis MyoB7, designed as ZmMyoBP7. In addition, the putative maize orthologs of Arabidopsis MyoB1-3 were also obtained for further analysis, designed as ZmMyoBP1-3. Domain analysis using the SMART database indicated that all these proteins share a highly confident DUF593 (zein-binding) motif, and the majority of them also contain coiled-coil regions and transmembrane domains (Fig.6A). It is worth noting that no confidently predicted transmembrane helix exists in FL1 and ZmMyoBP7 according to the TMHMM program.

To avoid the interference of the potential transmembrane domains existing in these proteins, only the DUF593 domains were used for detecting the interaction with myosins XI in yeast. In agreement with our previous results (Wang *et al.*, 2012*a*), O1 could not directly interact with the DUF593 domain of FL1 (Fig.6B). Moreover, unlike the Arabidopsis XI-I, neither ZmMyoBP7 nor ZmMyoBP1 could interact with O1 in the Y2H assay. This discrepancy may be caused by the functional divergence of paralogs O1/ZmMyo11G1 and ZmMyo11G2 within subgroup XI-I (Wang *et al.*, 2012*a*). However, similar to Arabidopsis XI-K, ZmMyo11E3 (XI-K ortholog) could interact with ZmMyoBP1-3 but not with FL1 and ZmMyoBP7 (Fig.6C). Taken together, the results show that the interaction between myosins XI and the DUF593-containing proteins is isoform-specific and probably conserved among different plants.

## Conclusions

In the current study, we developed a novel method for the isolation of highly purified and intact PBs in maize endosperm by a combination of several rounds of discontinuous and continuous sucrose-density gradient centrifugation. Using HPLC-MS, we have provided the first quantitative proteomic analysis of zein PBs, which contains 1756 proteins that fall into five major categories: metabolic pathways, biosynthesis of secondary metabolites, ribosomes, protein processing in the endoplasmic reticulum, and oxidative phosphorylation. This unexpectedly large number of proteins identified at 20 DAP might progressively decrease when compared to later stage PBs, such as 25 DAP, which might provide new insights into the key factors involved in PB biogenesis. In-depth analysis indicates that: (1) ribosomes and ER membranes (probably derived from rough ER), as well as the cytoskeleton, are most likely the peripheral compartment of the maize endosperm PB; (2) it is likely conserved that zein mRNAs are also transported and localized to the PB–ER subdomain; and (3) ER chaperones are essential for zein folding, quality control, and PB assembly. Surprisingly, there is no direct interaction between O1 and FL1 although both play essential roles in PB formation. We suggest that the interaction between myosins XI and DUF593-containing proteins is isoform-specific.

By combining all the results, we propose a roadmap for zein mRNAs transport, translation, and assembly into PBs in maize endosperm cells. After exporting from the nucleus to the cytosol, zein mRNAs are bound by the RNP complexes through their zipcode regions and are then jointly transported to the ER, probably by the actin-based actomyosin system (Hamada *et al.*, 2003). Different types of zeins are temporally synthesized on the ER membrane-bound ribosomes and enter into the secretory pathway due to their N-terminal signal peptides. In spite of the lack of ER retention signals, zeins are retained in the ER lumen by some unknown mechanism, most likely aided by ER chaperones. γ-zeins are deposited first and provide a scaffold for PB formation. As PBs are expanded by β-zeins, α- and δ-zeins are subsequently sequestered and filled into the PB core. During this process, O1 affects ER morphology and motility through binding the C-terminus of ZmHIP, whose N-terminal TPR domain interacts with an ER-anchored HSP70, and finally influences PB biogenesis (Wang *et al.*, 2012*a*).

## Supplementary data

Supplementary data can be found at *JXB* online.

Figure S1. Silver-stained analytical SDS gels of maize kernel proteins at 20 DAP before and after using the new protocol.

Figure S2. Western blotting for determining the presence of PBs and ER using the buffers with or without Mg^2+^.

Table S1. A list of primers used in this study.

Table S2. Distribution and density of major organelles from maize seeds in a 30–60% (w/w) continuous sucrose- density gradient.

Table S3. Full list of proteins present in the maize endosperm PB proteome.

Table S4. Subcellular localization analysis of the 550 proteins only present in the intact PBs.

Table S5. Proteins potentially involved in ribosome biogenesis.

Table S6. ER-associated proteins present in maize PBs.

Table S7. Putative RNA-binding proteins involved in prolamine RNA transport and localization in maize and rice.

Table S8. Predicted membrane-spanning proteins identified in maize PBs.

Dataset S1. The raw MS proteomics data of this study.

Dataset S2. GO classification of the proteins identified in the three maize endosperm PB proteomes (*P*-value <0.05, FDR<0.05).

Dataset S3. KEGG annotation of the proteins identified in the three maize endosperm PB proteomes (*P*-value <0.05, FDR<0.05).

Supplementary Data
