# Supplementary material for: Comprehensive proteomic analysis of developing protein bodies in maize (Zea mays) endosperm provides novel insights into its biogenesis
Source: J Exp Bot. 2016 Oct 27;67(22):6323–35. doi: 10.1093/jxb/erw396 (PMC5181578; doi:10.1093/jxb/erw396)
Supplement: Supplementary Data [file supp_67_22_6323__index.html]

Comprehensive proteomic analysis of developing protein bodies in maize (Zea mays) endosperm provides novel insights into its biogenesis — Comprehensive proteomic analysis of developing protein bodies in maize (Zea mays) endosperm provides novel insights into its biogenesis — Supplementary Data 

# Comprehensive proteomic analysis of developing protein bodies in maize (*Zea mays*) endosperm provides novel insights into its biogenesis

## Supplementary Data

Data files

- supplementary\_figures\_S1\_S2.pdf - Supplementary Data
- supplementary\_tables\_S1\_S8.xls - Supplementary Data
- supplementary\_dataset\_S1.xls - Supplementary Data
- supplementary\_dataset\_S2.xls - Supplementary Data
- supplementary\_dataset\_S3.xls - Supplementary Data
