# Supplementary material for: Comprehensive proteomic analysis of developing protein bodies in maize (Zea mays) endosperm provides novel insights into its biogenesis
Source: J Exp Bot. 2016 Oct 27;67(22):6323–35. doi: 10.1093/jxb/erw396 (PMC5181578; doi:10.1093/jxb/erw396)
Supplement: Supplementary Data [file supp_erw396_supplementary_figures_S1_S2.pdf]

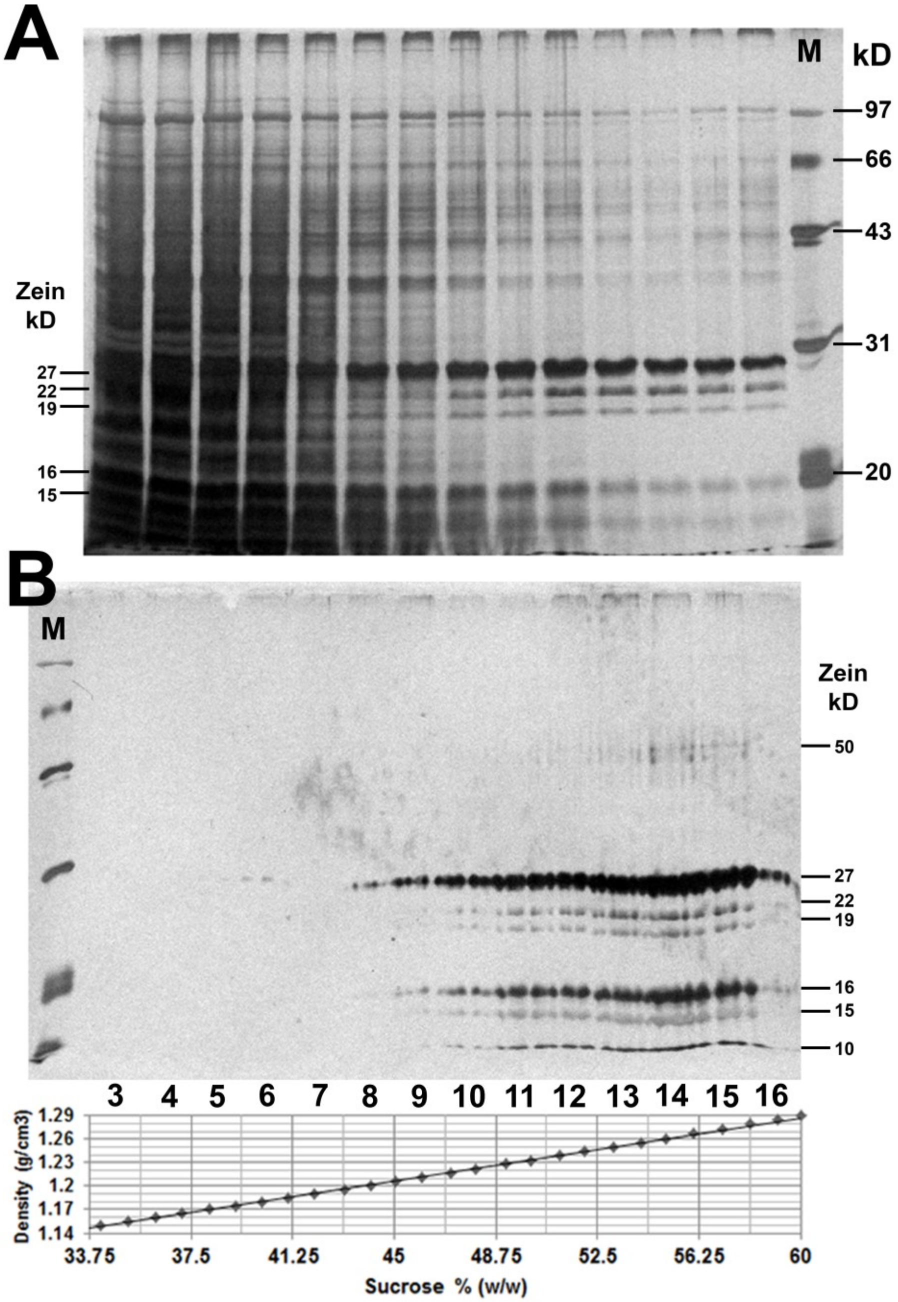

Supplemental Figure 1 Silver-stained analytical SDS gel of maize 20 DAP kernel proteins distributed in continuous fractions subfractionated from the 30-60% (w/w) continuous sucrose-density gradient, before (A) and after using the new protocol (B).

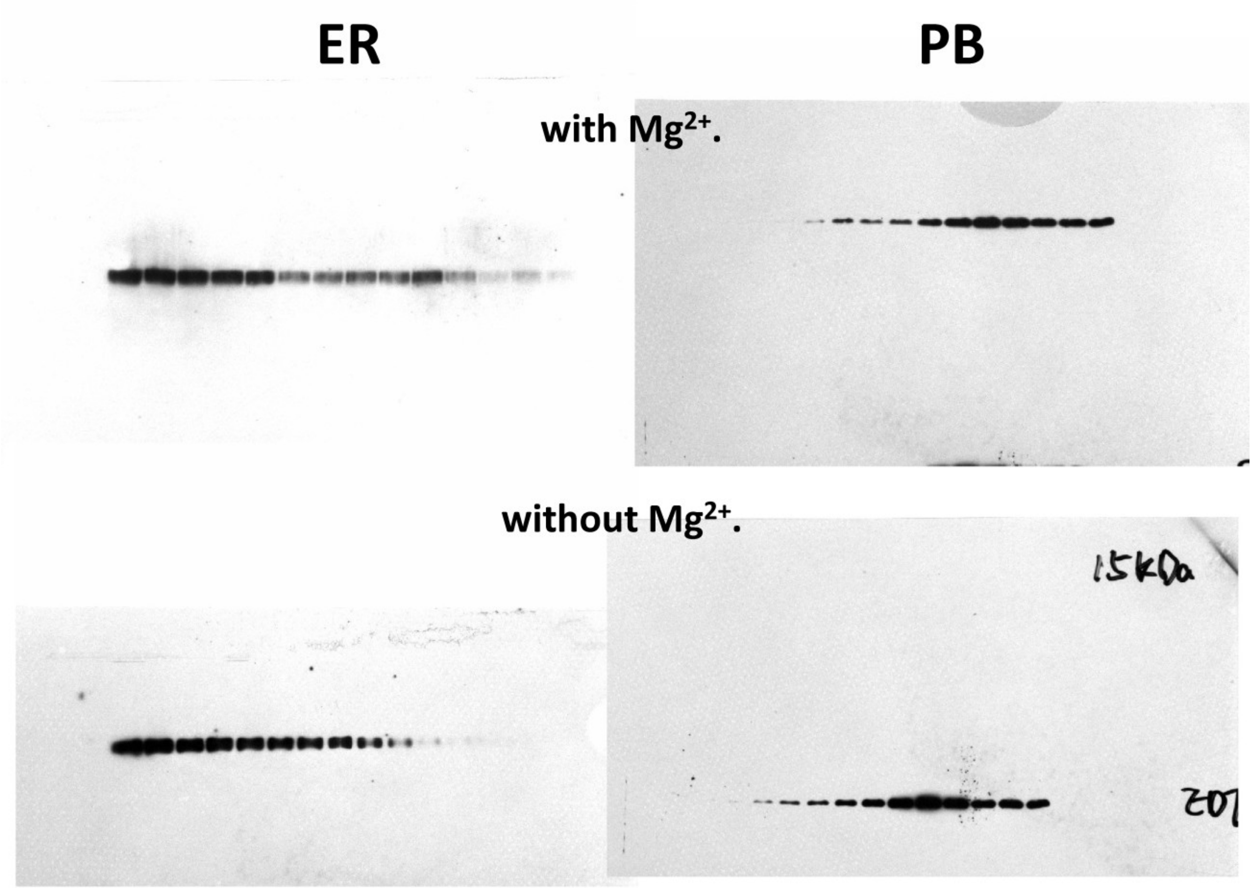

Supplemental Fig. 2 Western blotting for determining the presence of PBs and ER using the buffers with or without Mg^{2+}.
